# Tannin-rich strawberry vines fermented with lactic acid bacteria improve growth performance in Hu sheep

**DOI:** 10.3389/fvets.2025.1666125

**Published:** 2025-10-13

**Authors:** Wenguang Lu, Zimo Zhang, Zhigang Zhu, Yusen Li, Zhenyu Fang, Siqi Wang, Shuying Zhou, Ling Zhang, Yunhua Zhang, Lijuan Chen

**Affiliations:** Anhui Agricultural University, Hefei, China

**Keywords:** strawberry vines, lactic acid bacteria, anti-nutritional factors, Hu sheep, growth performance

## Abstract

**Background:**

Strawberry vines are nutrient-rich but contain high levels of tannins and have a moisture content of up to 70%, making ensiling a crucial strategy for preserving their nutritional value.

**Methods:**

Two lactic acid bacteria (LAB) strains, designated Z and R, were isolated from strawberry vines. Fresh strawberry vines were ensiled in three groups: two experimental groups inoculated with strains Z and R (1.0 × 10^6^ CFU/g fresh weight) and a control group (CK) treated with physiological saline. Samples were collected on days 1, 3, 5, 7, 15, 30, and 45 of fermentation for analysis. The LAB-treated silage was subsequently incorporated into Hu sheep diets to evaluate its effects on growth performance.

**Results:**

The identification results indicated that strain Z was *Lactiplantibacillus plantarum* and strain R was *Lactococcus lactis*. Both treatment groups exhibited significantly higher crude protein (CP) content (*p* < 0.05) and markedly lower NH_3_-N content (*p* < 0.05) compared to the CK group. Notably, supplementation with *L. plantarum* significantly reduced tannin content in strawberry vines (*p* < 0.05) compared to other two groups. By day 3, the pH values in the treatment groups were significantly lower than those in the CK group (*p* < 0.05), with *L. plantarum* treatment group showing significantly lower pH than *L. lactis* group throughout days 3 to 15 (*p* < 0.05), indicating more rapid silage stabilization. In terms of fermentation quality, lactic acid (LA) and acetic acid (AA) contents in the treatment groups were significantly higher than in the CK group (*p* < 0.05). Microbial community analysis demonstrated that both treatment groups effectively suppressed the growth of harmful microorganisms at both the phylum and genus levels, with *Lactiplantibacillus* genus abundance reaching 54.18% in *L. plantarum* group compared to only 0.98% in CK by day 3. Furthermore, when used as roughage for Hu sheep, LAB-fermented strawberry vine silage significantly improved average daily gain (ADG) (*p* < 0.05) and enhanced the apparent digestibility of dry matter (DM), neutral detergent fiber (NDF), and CP (*p* < 0.05). Inclusion of strawberry vine silage fermented by *L. plantarum* markedly reduced the relative abundances of the predominant ruminal genera *Clostridium* and *Ercella*.

**Conclusion:**

LAB supplementation significantly improved the silage quality of strawberry vines by effectively inhibiting putrefactive processes during the late fermentation stage of high-moisture silage. Moreover, the use of strawberry vine silage as feed markedly enhanced the growth performance in Hu sheep.

## Highlights

- This study represents the first attempt to use strawberry vines as feed.
- Anaerobic fermentation using LAB reduces the tannin content in strawberry vines.
- The addition of two LAB strains significantly increased organic acid content.
- The two LAB strains effectively preserved the nutrients of strawberry vines.
- Strawberry vines silage effectively improves the growth performance in Hu sheep.

## 1 Introduction

A sufficient supply of high-quality roughage is essential for the sustainable development of herbivorous livestock industries. However, China currently faces a shortage of nearly 50 million tons of premium roughage, highlighting the need to explore alternative resources (1). The efficient utilization of crop straw offers a promising solution for producing high-quality roughage. Although China generates approximately 800 million tons of crop straw annually, only about 20% is utilized in ruminant farming owing to its low nutritional value and poor palatability (2).

Strawberry (*Fragaria* genus, *Rosaceae* family), a perennial herbaceous plant, is rich in bioactive and nutritional compounds, such as anthocyanins, flavonols, ellagitannins, folic acid, and various vitamins. These constituents contribute to antioxidant activity, immune system enhancement, anti-aging effects, and cholesterol reduction. Strawberry vines, which contain CP, ether extract (EE), vitamin C, and trace minerals, exhibit good palatability and represent a source of high-quality forage. To date, relatively few studies have investigated strawberry vines, and their potential has not been effectively utilized. Following strawberry production, the residual by-product—the strawberry vines—remains unprocessed, resulting in environmental damage. Harvested during the rainy season in April and May in southern China, fresh strawberry vines typically exhibit a moisture content of approximately 70%, rendering natural drying impractical for preserving their nutritional quality. Ensiling offers an effective solution for nutrient preservation and the production of high-quality silage. This approach not only meets the demand for premium forage among herbivores but also helps reduce agricultural waste pollution, thereby supporting sustainable agro-pastoral development. Utilizing strawberry vine feed can mitigate environmental impacts, support the sustainable and circular development of the strawberry industry, and provide a substantial source of roughage for ruminant production.

Silage fermentation depends on LAB naturally present on fresh crops. These bacteria utilize water-soluble carbohydrates (WSC) under anaerobic conditions to produce LA, thereby creating an acidic environment that inhibits the growth of spoilage microorganisms (3). In the initial phase of ensiling, LAB dominates by metabolizing WSC into organic acids, which suppress the proliferation of harmful microbes (4–6). However, the competitive dynamics between LAB and other microorganisms are influenced by factors such as moisture content, airtightness, and the physiological characteristics of the LAB strains involved. The ability of LAB to rapidly adapt to the ensiling environment and reduce pH is crucial for achieving high-quality silage.

High-moisture silage (moisture content >70%) is susceptible to clostridial fermentation, which leads to the production of butyric acid and a consequent decline in silage quality (7). Excessive moisture dilutes WSC and LAB, hindering a rapid decline in pH (8). This environment further promotes clostridial activity, resulting in increased DM losses, protein hydrolysis, and butyrate accumulation. These factors collectively reduce feed digestibility, nitrogen utilization, and intake (9). Field wilting is a commonly used strategy to reduce moisture content; however, it carries risks such as physical losses, respiratory consumption of WSC, dependence on weather conditions, and mold contamination. As an alternative, additives can modulate microbial fermentation, suppress harmful microbes, and improve high-moisture silage quality. Different silage materials contain varying levels of anti-nutritional factors, such as tannins, which can affect the activity of LAB during ensiling and, consequently, influence silage quality (10). The tannin content of strawberry vines exceeds 2%, classifying them as a high-tannin silage material. Therefore, exploring indigenous LAB from strawberry vines could be beneficial for improving their silage quality. To address the challenges associated with the high moisture content and tannin-rich composition of strawberry vines, this study isolated LAB strains from strawberry vines and employed them for ensiling. The fermented silage was subsequently fed to Hu sheep to evaluate the effects of native LAB on the fermentation quality of high-moisture strawberry vines silage and its effectiveness in ruminant production.

## 2 Materials and methods

### 2.1 Experimental materials

Fresh strawberry vines were harvested in Changfeng County (117.23°N,32.12°E), Hefei City, Anhui Province, China. The strawberry vines used in the experiments were picked at the end of April.

The nutritional composition of the raw strawberry vines material is presented in the Table 1. The DM content of strawberry vines raw materials was 21.93%. On a DM basis, the composition included 11.70% CP, 16.85% EE, 29.71% NDF, 18.69% acid detergent fiber (ADF), and 14.52% WSC.

**Table 1:** Chemical composition of raw strawberry vines material.

Item	Value
DM (FM%)	21.93
CP (DM%)	11.70
EE (DM%)	16.85
NDF (DM%)	29.71
ADF (DM%)	18.69
WSC (DM%)	14.52

DM, dry matter; FM, fresh weight; CP, crude protein; EE, ether extract; NDF, neutral detergent fiber; ADF, acid detergent fiber; WSC, water-soluble carbohydrates.

### 2.2 Experimental design and sampling

#### 2.2.1 Isolation of LAB from strawberry vines

The isolation and identification of LAB shall refer to the Chinese national standard GB 4789.35–2023 (11). A 10 g sample of fresh strawberry vines was placed in a conical flask containing 90 mL of sterile saline solution for extraction. The resulting extract was serially diluted to concentrations of 10^−1^, 10^−2^, 10^−3^, and 10^−4^ under aseptic conditions. Aliquots from each dilution were spread onto De Man, Rogosa and Sharpe (MRS) agar plates using a sterile spreader and incubated at 37 °C for 24 h. Colonies exhibiting differences in morphology, size, and color were selected and repeatedly streaked onto fresh MRS agar plates for purification. Pure strains were preserved in 20% (v/v) glycerol at −80 °C.

#### 2.2.2 Silage preparation

Bacterial suspensions (strains Z and R) in the logarithmic growth phase were centrifuged at 4 °C for 3 min. The resulting pellets were washed three times with phosphate-buffered saline (PBS) to prepare the inoculants. Fresh strawberry vines were cut into 2–3 cm segments and mixed with the LAB inoculants under vacuum sealing. Three treatments were established as follows:

- CK group: Fresh strawberry vines + physiological saline (in a volume equivalent to that of the LAB inoculants).
- Treatment I group: Fresh strawberry vines + *L. plantarum* (strain Z, 1.0 × 10^6^ CFU/g fresh weight).
- Treatment II group: Fresh strawberry vines + *L. lactis* (strain R, 1.0 × 10^6^ CFU/g fresh weight).

The mixtures were homogenized, packed into silage bags (400 g per bag), vacuum-sealed, and stored in darkness at room temperature. Triplicate samples from each treatment were collected on days 1, 3, 5, 7, 15, 30, and 45 of ensiling for subsequent analysis.

#### 2.2.3 Feeding trial

Fresh strawberry vines were cut into 2–3 cm segments and ensiled with *L. plantarum* and *L. lactis* at a concentration of 1.0 × 10^6^ CFU/g. Each bag contained 50 kg of material, was vacuum-sealed, and stored for subsequent feeding to Hu sheep. Eighteen healthy male Hu sheep (initial body weight 27.15 ± 0.34 kg), with similar genetic backgrounds, were randomly assigned to three groups (*n* = 6 per group) as follows:

- CK group: Basal diet formulated according to the *Nutrient Requirements of Meat Sheep* (NY/T 816–2021).
- Treatment I group: Basal diet + 15% *L. plantarum*-fermented strawberry vines silage.
- Treatment II group: Basal diet + 15% *L. lactis*-fermented strawberry vines silage.

The composition and nutrients of Hu sheep feed are shown in Table 2. Sheep were housed in disinfected pens, dewormed, and acclimatized for 10 days prior to the commencement of the 60-day formal trial. A total mixed ration (TMR) was provided twice daily (at 08:00 and 16:00), with free access to water. Residual feed was collected and weighed daily to calculate dry matter intake (DMI). Body weights were recorded at both the start and end of the trial to determine the ADG and the feed-to-gain ratio (F/G). On day 60 of the trial, rumen fluid samples (5 mL) were collected following a 12-h fasting period for 16S rRNA sequencing to analyze microbial community structure. On day 60, feed and fecal samples were collected to assess apparent digestibility.

**Table 2:** Composition and nutritional content of the basal diet for Hu sheep.

Item	Groups		
	CK	I	II
Composition of components (%DM)			
Strawberry vines	0.00	15.00	15.00
Corn	17.00	17.00	17.00
Corn straw	55.00	42.00	42.00
Soybean meal	16.00	14.00	14.00
Bran	8.00	8.00	8.00
Premix	2.50	2.50	2.50
NaHCO_3_	0.70	0.70	0.70
Salt	0.80	0.80	0.80
Total	100.00	100.00	100.00
Nutritional composition			
DM	37.20	37.01	36.81
CP (%DM)	14.69	14.73	14.70
NDF (%DM)	44.62	40.40	42.22
ADF (%DM)	34.65	32.05	33.59
Ash (%DM)	7.31	6.42	6.70

DM, dry matter; CP, crude protein; NDF, neutral detergent fiber; ADF, acid detergent fiber.

### 2.3 Measurements

#### 2.3.1 LAB identification

Isolated strains were subjected to catalase testing, gas production assays from glucose fermentation, and carbohydrate fermentation assays. Genomic DNA was extracted from enriched cultures using the BIOMIGA DNA Extraction Kit following the manufacturer’s instructions. Polymerase Chain Reaction (PCR) amplification of the 16S rRNA gene was performed using universal primers (27F: 5′-AGRGTTYGATYMTGGCTCAG-3′; 1492R: 5′-RGYTACCTTGTTACGACTT-3′). The reaction mixture (50 μL) consisted of 10 μL of 5 × Fast Pfu Buffer, 2 μL of 2.5 mM dNTPs, 1 μL each of forward and reverse primers (5 μM), 0.5 μL of Fast Pfu Polymerase, 10 ng of template DNA, and ddH₂O. PCR conditions were as follows: initial denaturation at 98 °C for 2 min; 35 cycles of 98 °C for 10 s, 57 °C for 10 s, and 72 °C for 45 s; final extension at 72 °C for 5 min; followed by a hold at 4 °C. The amplified products were sequenced and analyzed via BLAST against the NCBI database (BLAST: Basic Local Alignment Search Tool) for species identification.

#### 2.3.2 Fermentation characteristics and tannin content

For pH measurement, 10 g of silage was mixed with 90 mL of distilled water, extracted at 4 °C for 24 h, and filtered through four layers of gauze. For organic acid analysis, 20 g of silage was homogenized with 180 mL of ultrapure water, filtered through quantitative filter paper and 0.45 μm cellulose ester membranes, and analyzed for LA and AA using high-performance liquid chromatography (HPLC), following the method described by Wang et al. (6). NH₃-N was quantified according to the method of Broderick and Kang et al. (12). The DM content of both silage and apparent digestibility was determined using the oven-drying method according to AOAC standards (13). The CP content of silage and apparent digestibility was analyzed using a fully automated Kjeldahl nitrogen analyzer following AOAC standards (13). Meanwhile, the NDF and ADF contents of silage and apparent digestibility were determined using the methods described by Van Soest et al. (14). EE was determined following GB/T 6433-2006 (15); and WSC was quantified using the Abbkine Soluble Sugar Assay Kit (Abbkine Scientific Co., Ltd., KTB1320). Tannin content was assessed using the Abbkine Tannin Content Assay Kit (Abbkine Scientific Co., Ltd., KTB1541).

Apparent nutrient digestibility was calculated using acid-insoluble ash as an internal marker, according to the following formula:


X=(1−N/M×K/L)×100%

Where *X* = apparent digestibility of the nutrient; *N* = acid-insoluble ash content in the feed; *M* = acid-insoluble ash content in the feces; *K* = nutrient content in the feces; *L* = nutrient content in the feed.

#### 2.3.3 Microbial diversity analysis

Bacterial DNA was extracted from silage and rumen fluid samples using the MagPure Tissue DNA LQ Kit (Magen, D6321-02). DNA concentration and integrity were assessed via NanoDrop 2000 spectrophotometry (Thermo Fisher Scientific, USA) and agarose gel electrophoresis. The V3–V4 hypervariable regions of the 16S rRNA gene were amplified using primers 343F (5′-TACGGRAGGCAGCAG-3′) and 798R (5′-AGGGTATCTAATCCT-3′), with reverse primers containing sample-specific barcodes. PCR products were purified using Agencourt AMPure XP beads (Beckman Coulter, A63880), quantified with the Qubit dsDNA Assay Kit (Thermo Fisher Scientific, Q32854), and sequenced on the Illumina NovaSeq 6000 platform (250 bp paired-end reads; OE Biotech Co., Shanghai, China).

### 2.4 Data processing and analysis

Data were processed using Excel 2019 and analyzed by one-way Analysis of Variance (ANOVA) followed by Duncan’s multiple range test in SPSS version 20.0. Results are presented as the mean ± standard deviation. Microbial diversity metrics were analyzed using the Personalbio GeneCloud platform (Shanghai Personal Biotechnology Co., Ltd., Shanghai, China). Statistical significance was defined at *p* < 0.05.

## 3 Results

### 3.1 Isolation and identification of LAB

To mitigate tannin-mediated inhibition of LAB activity, we isolated two LAB strains (designated Z and R) from strawberry vines. Their morphological characteristics, Gram staining results, and cellular morphology are detailed in Table 3, while their physiological and biochemical profiles are presented in Table 4. Carbon utilization assays revealed comparable metabolic capabilities between strains Z and R across most substrates, with the notable exception of salicin metabolism, which was unique to strain Z and absent in strain R. 16S rRNA gene sequencing, conducted via PCR amplification followed by BLAST alignment, confirmed 99.39% sequence homology between strain Z and *L. plantarum*, while strain R exhibited 99.57% homology with *L. lactis*. An integrative analysis of morphological and molecular data conclusively identified strain Z as *L. plantarum* and strain R as *L. lactis*.

**Table 3:** Morphological characteristics of isolated LAB strains.

Strain	Gram staining	Colony characteristics	Cell morphology
Z	G+	Circular, creamy-white colonies with smooth, flawless surfaces and entire margins	Rod-shaped
R	G+	Creamy-white, circular colonies with smooth, glossy surfaces and distinct, smooth margins	Coccoid

**Table 4:** Physiological and biochemical identification results of strains Z and R.

Test item	Result		Test item	Result	
	Z	R		Z	R
Gas production from glucose	−	−	Salicin	+	−
Cellobiose	+	+	Inulin (from chicory)	+	+
Melibiose	+	+	Esculin hydrolysis	+	+
Maltose	+	+	D-Ribose	+	+
Lactose	+	+	Xylose	+	+
Sorbitol	−	−	Arabinose	+	+
Mannitol	+	+	Inositol	−	−
Raffinose	+	+	Citrate utilization	−	−
Urea hydrolysis	−	−	Glucose-phosphate peptone water	−	−
Sodium hippurate hydrolysis	+	+	Ornithine decarboxylase	−	−

“+” indicates a positive result; “-” indicates a negative result.

### 3.2 Effect of LAB on the nutritional composition of strawberry vines silage

Table 5 presents the nutritional changes observed during the ensiling process of strawberry vines. The DM content in all groups decreased during the initial ensiling phase and stabilized after 15 days. At 7 days, Group I exhibited significantly higher DM content compared to both the CK group and Group II (*p* < 0.05). The WSC content in the experimental groups declined rapidly during the early stages of ensiling and was significantly lower than that of the CK group after 5 days (*p* < 0.05). Throughout the ensiling period, CP content declined across all groups; however, Group I experienced a slower rate of decrease. At 45 days, Group I maintained significantly higher CP content than both the CK group and Group II (*p* < 0.05). Group II exhibited a slower reduction in EE content at 15 and 30 days, maintaining significantly higher values than the other groups (*p* < 0.05); however, no significant differences were observed among groups at 45 days (*p* > 0.05). Both NDF and ADF contents decreased progressively during ensiling, with NDF showing a slower rate of decline. After 5 days, the CK group maintained significantly higher NDF and ADF contents compared to the experimental groups (*p* < 0.05).

**Table 5:** Effect of LAB on the nutrient composition of strawberry vines silage.

Item	Treatment (T)	Storage period (D/d)							*p*-value		
		1	3	5	7	15	30	45	T	D	T × D
DM, % FM	CK	21.76 ± 0.23a	21.54 ± 0.10a	20.52 ± 0.39b	20.09 ± 0.02Bc	19.86 ± 0.01Bc	19.77 ± 0.03Cc	19.23 ± 0.08 Bd	<0.001	<0.001	<0.001
	I	21.92 ± 0.04a	21.64 ± 0.12b	20.72 ± 0.20c	20.65 ± 0.17Ac	20.51 ± 0.08Ac	20.54 ± 0.12Ac	20.27 ± 0.12Ad			
	II	21.74 ± 0.16a	21.58 ± 0.22a	20.63 ± 0.27b	20.59 ± 0.23Ab	20.41 ± 0.20Abc	20.38 ± 0.03Bbc	20.12 ± 0.20Ac			
CP, % DM	CK	11.56 ± 0.03a	11.58 ± 0.01a	11.33 ± 0.08b	10.89 ± 0.05Cc	10.72 ± 0.01Cd	10.70 ± 0.03Cd	10.25 ± 0.05Ce	<0.001	<0.001	<0.001
	I	11.62 ± 0.08a	11.56 ± 0.06a	11.43 ± 0.01b	11.41 ± 0.08Ab	11.21 ± 0.11Ac	11.07 ± 0.01Ad	10.62 ± 0.02Ae			
	II	11.64 ± 0.03a	11.60 ± 0.06a	11.41 ± 0.08b	11.19 ± 0.05Bc	10.89 ± 0.06 Bd	10.77 ± 0.03Be	10.51 ± 0.09Bf			
EE, % DM	CK	15.76 ± 0.19a	15.45 ± 0.27a	14.40 ± 0.09b	14.38 ± 0.10b	12.67 ± 0.08Bc	12.58 ± 0.26ABc	12.12 ± 0.43d	<0.001	<0.001	<0.001
	I	15.79 ± 0.02a	15.19 ± 0.07b	14.37 ± 0.22c	13.61 ± 0.68d	12.35 ± 0.37Be	12.04 ± 0.14Bef	11.62 ± 0.24f			
	II	15.74 ± 0.20a	15.51 ± 0.11a	14.70 ± 0.35b	14.28 ± 0.10b	14.21 ± 0.90Ab	12.73 ± 0.45Ac	12.06 ± 0.38c			
NDF, % DM	CK	26.58 ± 0.33a	25.79 ± 0.11b	25.54 ± 0.10Ab	24.74 ± 0.06Ac	24.55 ± 0.05Acd	24.43 ± 0.21Ad	24.31 ± 0.06Ad	<0.001	<0.001	<0.001
	I	26.47 ± 0.15a	25.64 ± 0.38b	25.23 ± 0.14Bc	24.13 ± 0.15 Bd	23.71 ± 0.10Be	23.57 ± 0.11Bef	23.34 ± 0.08Bf			
	II	26.52 ± 0.14a	25.60 ± 0.35b	24.32 ± 0.14Cc	23.34 ± 0.37Cd	22.73 ± 0.02Ce	22.58 ± 0.07Ce	22.44 ± 0.06Ce			
ADF, % DM	CK	20.59 ± 0.33a	19.86 ± 0.03b	19.77 ± 0.02Ab	19.71 ± 0.02Ab	19.63 ± 0.12Abc	19.41 ± 0.08Ac	19.42 ± 0.10Ac	<0.001	<0.001	<0.001
	I	20.67 ± 0.36a	19.81 ± 0.01b	19.74 ± 0.13Ab	19.06 ± 0.17Bc	18.84 ± 0.02Bcd	18.77 ± 0.06Bcd	18.60 ± 0.01 Bd			
	II	20.24 ± 0.11a	19.74 ± 0.10b	19.61 ± 0.03Bb	18.67 ± 0.06Cc	18.64 ± 0.12Bc	18.29 ± 0.15Cd	18.27 ± 0.12Cd			
WSC, % DM	CK	13.64 ± 0.08a	12.39 ± 0.09b	12.27 ± 0.08Ab	11.48 ± 0.39Ac	10.70 ± 0.18Ad	9.46 ± 0.37Ae	8.68 ± 0.25Af	<0.001	<0.001	<0.001
	I	13.68 ± 0.26a	12.22 ± 0.19b	11.79 ± 0.13Bc	9.46 ± 0.29 Bd	9.30 ± 0.07 Bd	8.48 ± 0.36Be	7.62 ± 0.09Bf			
	II	13.67 ± 0.20a	12.39 ± 0.15b	10.17 ± 0.04Cc	9.56 ± 0.38 Bd	9.65 ± 0.27 Bd	8.60 ± 0.01Be	7.63 ± 0.20Bf			

DM, dry matter; FM, fresh weight; CP, crude protein; EE, ether extract; NDF, neutral detergent fiber; ADF, acid detergent fiber; WSC, water-soluble carbohydrates. Within the same row, values followed by different lowercase letters differ significantly (*p* < 0.05), while values with the same letter do not (*p* > 0.05). Similarly, within the same column, values followed by different uppercase letters indicate a significant difference (*p* < 0.05), while values with the same letter or without a letter indicate no significant difference (*p* > 0.05). The same notation applies to the tables below.

### 3.3 Effect of LAB on the fermentation quality and tannin content of strawberry vines silage

The fermentation quality and tannin content of ensiled strawberry vines are presented in Table 6. In Groups I and II, pH decreased rapidly during the early stages of ensiling, falling below 4 by days 5 and 7, respectively. From day 3 onward, both experimental groups maintained significantly lower pH values than the CK group (*p* < 0.05), with Group I showing significantly lower pH than Group II throughout days 3 to 15 (*p* < 0.05).

**Table 6:** Effects of LAB on the fermentation quality of strawberry vines silage.

Item	Treatment (T)	Storage period (D/d)							*p*-value		
		1	3	5	7	15	30	45	T	D	T × D
pH	CK	5.24 ± 0.03a	5.09 ± 0.03Ab	4.83 ± 0.06Ac	4.35 ± 0.04Ad	4.26 ± 0.03Ae	4.12 ± 0.04Af	3.73 ± 0.01Ag	<0.001	<0.001	<0.001
	I	5.10 ± 0.10a	4.08 ± 0.06Cb	3.86 ± 0.04Cc	3.76 ± 0.02Cd	3.71 ± 0.02Cde	3.69 ± 0.03Bde	3.65 ± 0.03Be			
	II	5.18 ± 0.07a	4.76 ± 0.03Bb	4.08 ± 0.05Bc	3.82 ± 0.01 Bd	3.76 ± 0.01 Bde	3.72 ± 0.02Be	3.61 ± 0.04Bf			
LA	CK	2.20 ± 0.03Be	2.27 ± 0.07Ce	2.58 ± 0.11 Bd	3.19 ± 0.06Bc	3.95 ± 0.02Cb	4.13 ± 0.07Ca	3.92 ± 0.06Bb	<0.001	<0.001	0.003
	I	2.47 ± 0.13Ac	2.56 ± 0.01Ac	3.88 ± 0.11Ab	3.87 ± 0.54Ab	4.81 ± 0.09Aa	4.87 ± 0.08Aa	4.69 ± 0.20Aa			
	II	2.28 ± 0.17ABe	2.44 ± 0.01Be	2.99 ± 0.58 Bd	3.52 ± 0.05ABc	4.24 ± 0.10Bb	4.70 ± 0.03Ba	4.46 ± 0.05Aab			
AA	CK	1.58 ± 0.11f	1.65 ± 0.01f	1.92 ± 0.07Be	2.41 ± 0.08Cd	2.71 ± 0.04Bc	3.39 ± 0.04Ba	3.24 ± 0.05Cb	<0.001	<0.001	<0.001
	I	1.51 ± 0.13 g	1.69 ± 0.02f	2.56 ± 0.12Ae	3.08 ± 0.04Ad	3.46 ± 0.06Ac	3.74 ± 0.03Aa	3.60 ± 0.02Ab			
	II	1.52 ± 0.08e	1.60 ± 0.09e	1.90 ± 0.05 Bd	2.83 ± 0.03Bc	3.38 ± 0.04Ab	3.69 ± 0.04Aa	3.28 ± 0.04Bb			
NH_3_-N	CK	1.81 ± 0.02f	1.96 ± 0.02e	2.06 ± 0.04Ad	2.14 ± 0.03Ad	2.38 ± 0.07Ac	2.62 ± 0.08Ab	2.86 ± 0.06Aa	<0.001	<0.001	<0.001
	I	1.78 ± 0.04e	1.92 ± 0.07d	1.98 ± 0.01Bcd	1.94 ± 0.08 Bd	2.05 ± 0.03Cc	2.18 ± 0.07Cb	2.35 ± 0.04Ca			
	II	1.78 ± 0.05f	1.90 ± 0.09e	1.93 ± 0.02 Bd	2.04 ± 0.04ABd	2.18 ± 0.08Bc	2.41 ± 0.08Bb	2.69 ± 0.06Ba			
Tannin	CK	2.54 ± 0.12a	2.21 ± 0.05Bb	2.03 ± 0.06Bc	1.83 ± 0.04Ad	1.53 ± 0.14Ae	1.43 ± 0.14Ae	1.20 ± 0.04Bf	0.006	<0.001	<0.001
	I	2.52 ± 0.09a	1.61 ± 0.03Cb	1.48 ± 0.05Cc	1.26 ± 0.08 Bd	1.17 ± 0.01 Bde	1.13 ± 0.06Be	1.01 ± 0.04Cf			
	II	2.51 ± 0.07a	2.40 ± 0.06Aa	2.40 ± 0.06Aa	1.74 ± 0.07Ab	1.70 ± 0.07Ab	1.48 ± 0.03Ac	1.46 ± 0.09Ac			

LA, lactic acid; AA, acetic acid; NH3-N, ammonium nitrogen.

This study observed increased LA and AA concentrations, with Groups I and II demonstrating more rapid LA accumulation, which became significantly higher than that of the CK group by day 3 (*p* < 0.05). Throughout the ensiling process, Group I consistently maintained higher LA levels than the other groups, while its AA content was significantly greater than both CK and Group II from day 5 onward (*p* < 0.05). In the CK group, NH_3_-N increased rapidly during the early ensiling period, significantly exceeding levels in the experimental groups after 5 days (*p* < 0.05). From day 7 onward, Group I exhibited significantly lower NH_3_-N concentrations than Group II (*p* < 0.05).

After 45 days of ensiling, Group I exhibited significantly lower tannin content compared to both the CK and Group II (*p* < 0.05), whereas Group II did not improve tannin degradation efficiency.

### 3.4 Effect of LAB on the microbial community structure of strawberry vines silage

#### 3.4.1 Analysis of microbial community composition at the phylum level

**Figure 1:**
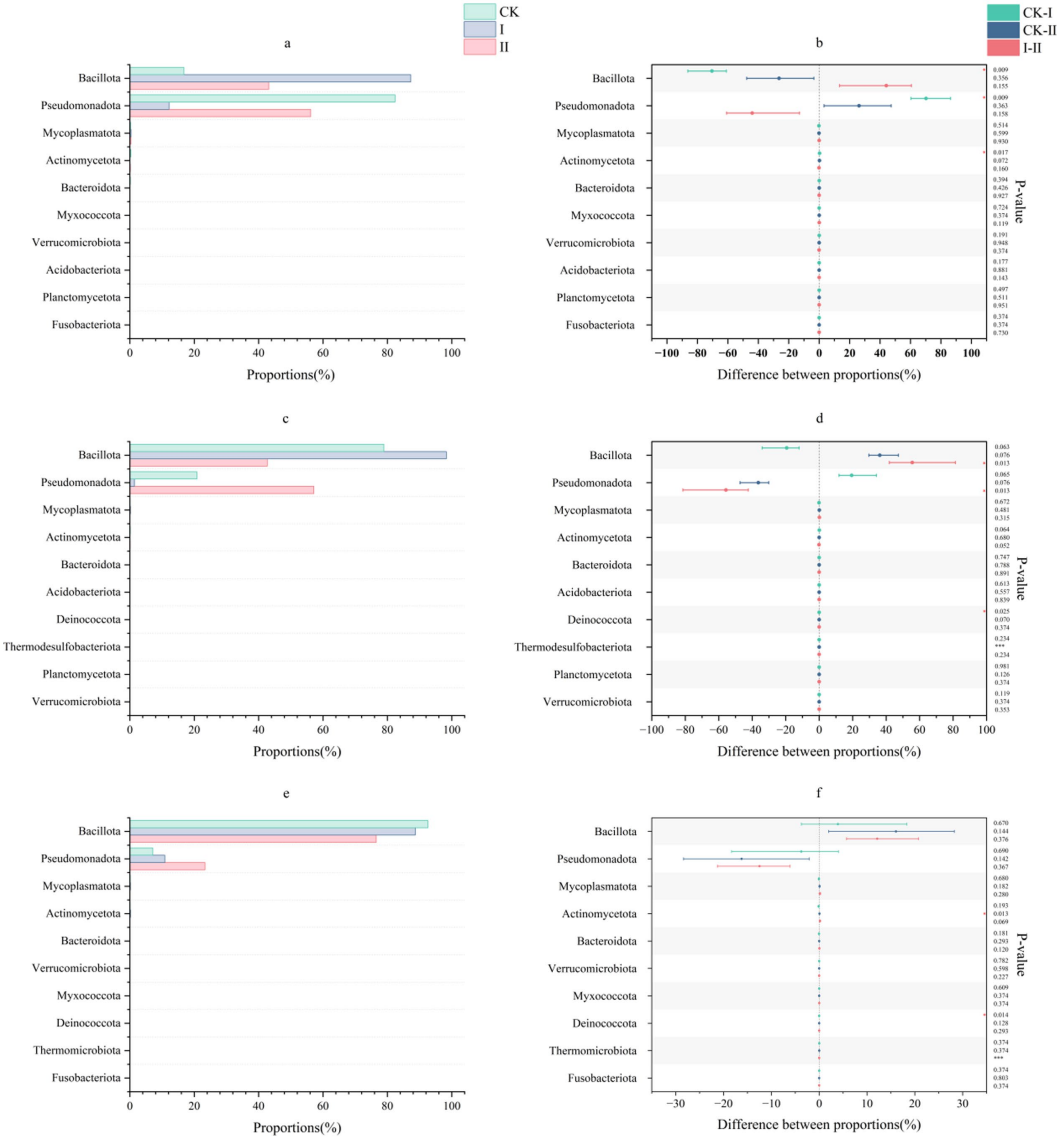
Effect of LAB on the microbial phylum level of strawberry vines silage over time. **(a,c,e)** Phylum-level microbial composition of strawberry vines silage on days 3, 7, and 45. **(b,d,f)** Differences in phylum-level microbial communities of strawberry vines silage on days 3, 7, and 45.

The phylum-level microbial community structure in strawberry vines silage is illustrated in Figure 1. In the present study, phylum-level community analysis revealed that *Bacillota* and *Pseudomonadota* predominated in the CK group and Group II during the early stages of ensiling, with *Pseudomonadota* abundance reaching 82.39% in the CK group and 56.19% in Group II. The abundance of *Bacillota* progressively increased with the duration of ensiling in these groups. Notably, *Bacillota* abundance in Group I reached 87.26% by day 3, demonstrating the superior adaptability of *L. plantarum* to ensiling conditions.

#### 3.4.2 Analysis of microbial community composition at the genus level

**Figure 2:**
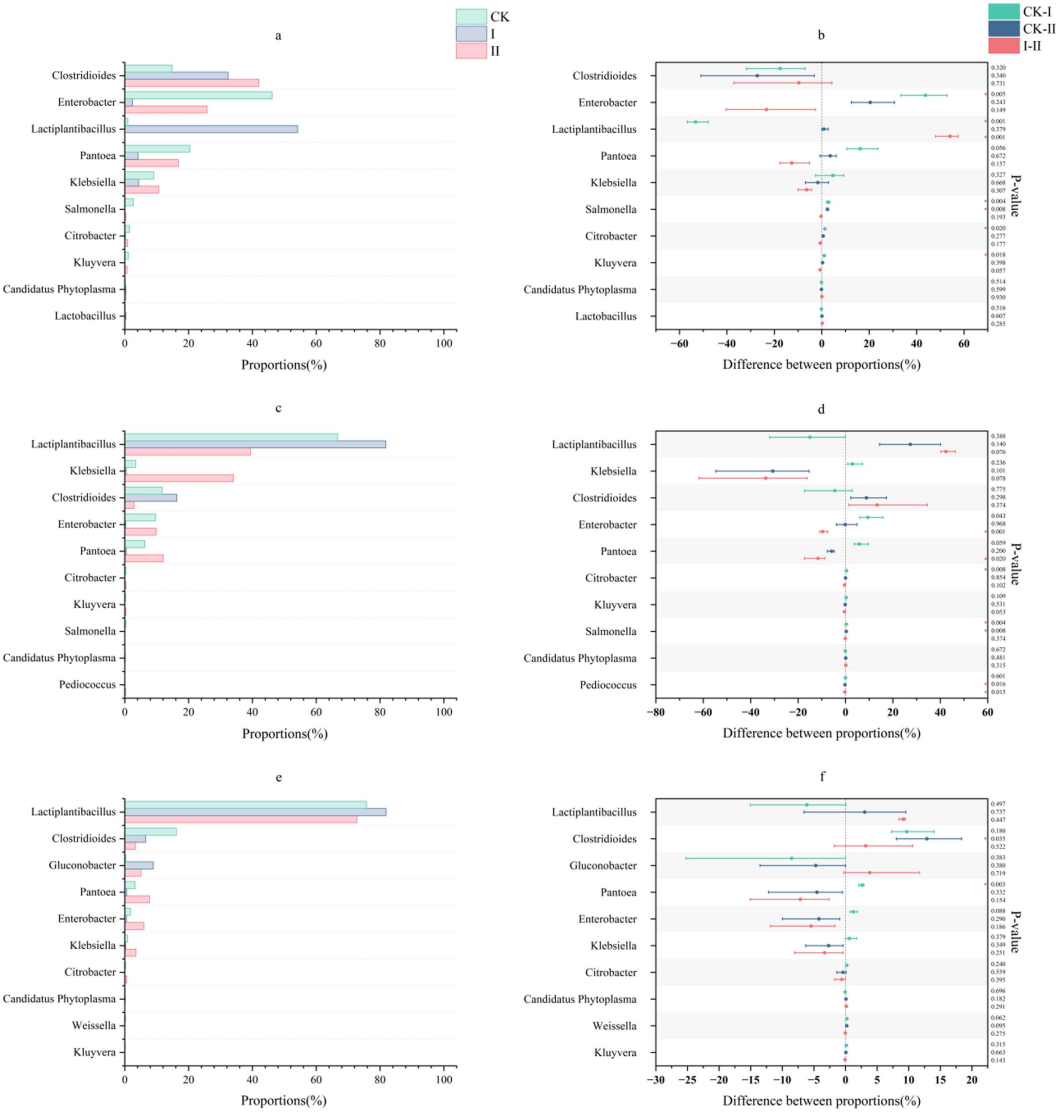
Effect of LAB on the microbial genus level of strawberry vines silage over time. **(a,c,e)** Genus-level microbial composition of strawberry vines silage on days 3, 7, and 45. **(b,d,f)** Differences in genus-level microbial communities of strawberry vines silage on days 3, 7, and 45.

The genus-level microbial community structure in strawberry vines silage is depicted in Figure 2. At the genus level, the CK group during the early stages of ensiling showed relative abundances of 14.83% for *Clostridioides*, 46.20% for *Enterobacter*, and 20.38% for *Pantoea*. Group II initially exhibited 42.03% *Clostridioides*, 25.75% *Enterobacter*, and 16.80% *Pantoea*, without significant *Lactococcus* presence. Notably, the abundance of *Klebsiella* in Group II increased from 10.71 to 34.09% by day 7. In contrast, Group I was dominated by *Lactiplantibacillus* (54.18%) and *Clostridioides* (32.38%) during the early stages of ensiling. Throughout the ensiling process, the abundance of *Lactiplantibacillus* progressively increased across all groups, while the populations of *Clostridioides*, *Enterobacter*, and *Pantoea* declined.

### 3.5 Effect of strawberry vines silage on production performance in Hu sheep

The effects of strawberry vines silage on production performance and apparent digestibility in Hu sheep are presented in Table 7. In this study, supplementation with LAB-fermented silage resulted in a significantly higher final body weight in Group I compared to the CK group (*p* < 0.05). Both experimental groups demonstrated significantly improved ADG and reduced F/G relative to the CK group (*p* < 0.05), although no significant differences were observed in DMI.

**Table 7:** Effect of strawberry vines silage on the production performance and apparent digestibility of Hu sheep.

	CK	I	II	*p*-value
Production performance				
Final weight/kg	68.60 ± 3.98b	71.55 ± 1.14a	71.31 ± 1.56ab	0.069
DMI/g	899.67 ± 64.35	903.48 ± 56.11	905.77 ± 77.15	0.917
ADG/g	118.57 ± 32.61b	143.23 ± 10.20a	141.67 ± 11.43a	0.059
F/G	7.59 ± 0.05a	6.31 ± 0.06b	6.39 ± 0.03b	<0.001
Apparent digestibility				
DM/%	61.83 ± 0.89c	65.08 ± 1.75b	68.34 ± 0.87a	0.002
NDF/%	54.26 ± 0.28b	56.66 ± 0.23a	55.68 ± 1.50ab	0.044
ADF/%	57.36 ± 0.43	56.58 ± 1.37	56.78 ± 0.75	0.593
CP/%	55.19 ± 1.32b	60.57 ± 1.33a	59.10 ± 0.13a	0.002

DMI, dry matter intake; ADG, average daily gain; F/G, feed-to-gain ratio; DM, dry matter; NDF, neutral detergent fiber; ADF, acid detergent fiber; CP, crude protein.

In the present study, LAB-fermented silage significantly improved the apparent digestibility of DM, NDF, and CP in Hu sheep (*p* < 0.05), although no significant enhancement was observed in ADF digestibility.

### 3.6 Effect of strawberry vines silage on the rumen microbial community structure in Hu sheep

**Figure 3:**
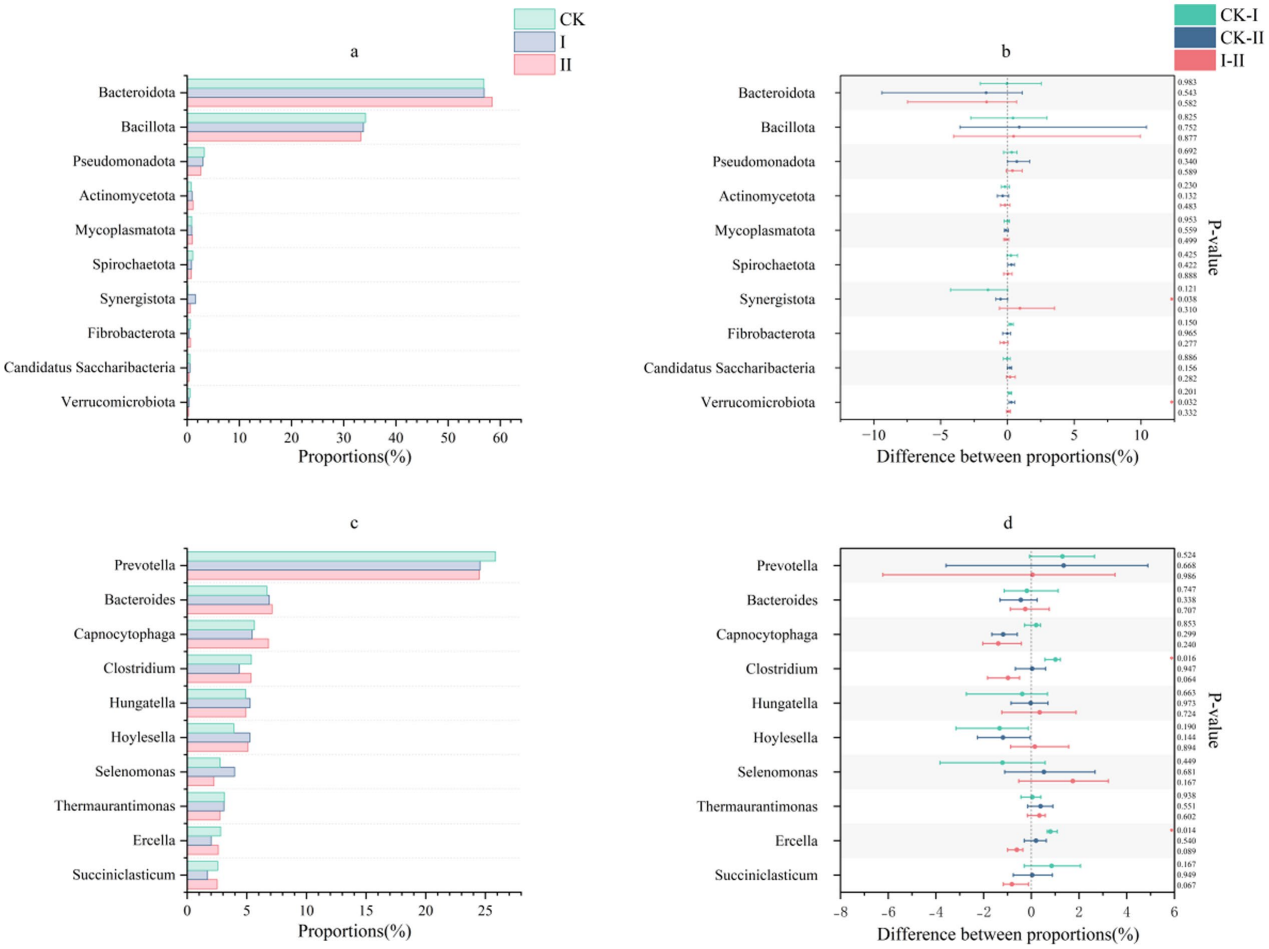
Effects of strawberry vines silage on the rumen microbiota composition in Hu sheep at the phylum and genus levels. **(a,b)** Composition and differences in the rumen fluid microbiota at the phylum level. **(c,d)** Composition and differences in the rumen fluid microbiota at the genus level.

The phylum-level composition of ruminal microbiota in Hu sheep is illustrated in Figures 3a,b. In the present study, *Bacteroidota* and *Bacillota* were the dominant phyla across all groups, with no significant intergroup differences observed. Genus-level microbial profiles of ruminal fluid, presented in Figures 3c,d, reveal predominant genera including *Prevotella*, *Bacteroides*, *Capnocytophaga*, and *Clostridium*. Compared to the CK group, both experimental groups exhibited a reduction in *Prevotella* abundance, although no inter-experimental group differences were observed.

## 4 Discussion

LAB constitutes one of the critical determinants influencing silage quality (16). LAB can effectively improve the fermentation quality of silage by rapidly lowering the pH of the feed and inhibiting the reproduction of harmful microorganisms. Their adaptability to the silage environment is modulated by the chemical composition of the feedstock, which is particularly affected by anti-nutritional factors such as tannins that inhibit LAB activity. Boonaert et al. reported that the physiological and biochemical characteristics of LAB isolated from plant surfaces may correlate with host plant chemistry (17), suggesting that epiphytic LAB strains exhibit enhanced environmental adaptation to their native host plants. This implies that host-specific epiphytic LAB inoculants could optimize silage quality when applied to homologous plant materials. Ke et al. demonstrated that tannins suppress LAB metabolic activity and delay their proliferation during ensiling (18). Notably, the high tannin content in the strawberry vines used in the present study likely impeded the early-phase pH reduction in the silage system. To mitigate tannin-mediated inhibition of LAB activity, thereby effectively retaining the nutrients in strawberry vines and improving their palatability for use in livestock production, we isolated two LAB strains from strawberry vines, one being *L. lactis* and the other *L. plantarum*.

Zhu et al. emphasized the pivotal role of carbon source utilization capacity in LAB performance (19), noting that strains with broad metabolic versatility can rapidly acidify the silage environment (through pH reduction), suppress the growth of undesirable microorganisms, and preserve the nutritional integrity of the feedstock, thereby enhancing silage quality. *L. plantarum* possesses a unique ability to metabolize salicin compared to *L. lactis*. The phenylpropanoid pathway, mediated by phenylalanine ammonia-lyase (PAL), produces salicylic acid and tannins. Ibrahim et al. reported a synergistic relationship between salicylic acid and tannin biosynthesis (20), whereby increased salicylic acid levels enhance PAL and chalcone synthase (CHS) activities, thereby promoting the synthesis of trans-cinnamic acid derivatives, flavonoids, and tannins in plants. Given the high tannin content in strawberry vines, elevated salicylic acid concentrations were hypothesized in this system. Strain Z’s ability to metabolize salicylic acid may underlie its rapid pH-lowering effect observed during ensiling.

Borreani et al. demonstrated that aerobic respiration during ensiling results in varying degrees of DM loss in forage (9), with lower WSC content exacerbating this depletion. In the present study, the relatively high WSC content in strawberry vines provided sufficient substrate for LAB fermentation, which rapidly lowered pH and effectively preserved DM content. After 45 days of ensiling, both experimental groups showed significantly lower WSC content and higher DM content compared to the CK group, consistent with the findings of Liu et al. (21). Moreover, both experimental groups showed significantly higher CP content than the CK group at 45 days, indicating that LAB additives effectively inhibited protein degradation, in agreement with the conclusions of Khota et al. (22). Han et al. reported that post-harvest plant lipases decompose lipids into glycerol and free fatty acids, with high moisture content accelerating this process (23). In the present study, the rapid pH reduction observed in Group I may also be associated with LA production via glycerol metabolism mediated by *L. plantarum*. Zhang et al. suggested that LAB metabolism during ensiling degrades NDF and ADF (24). The present study observed similar degradation patterns for NDF and ADF during fermentation, with experimental groups showing significantly lower values than the CK group (*p* < 0.05).

The pH reduction in silage primarily results from organic acid production through LAB metabolism during fermentation. Ma et al. demonstrated that silage additives can effectively lower pH, with this reduction enhancing aerobic stability and facilitating long-term preservation (25). Experimental Groups I and II exhibited significantly LA and AA contents compared to the CK group, accompanied by markedly lower pH values. Dai et al. reported that hydrolyzable tannins in plants possess unstable structures that are susceptible to enzymatic degradation by tannase or lyase, potentially generating gallic acid (26), which may oxidize into stronger acids such as oxalic acid. Additionally, salicin present in strawberry vines may undergo microbial hydrolysis to form salicylic acid during fermentation, simultaneously serving as a carbon source for LAB. The early addition of *L. plantarum* in Group I accelerated tannin degradation into acidic compounds such as gallic acid, thereby further promoting pH reduction. LA and AA in silage, predominantly produced through LAB metabolism, serve as crucial indicators for evaluating fermentation quality (27). In this study, LA and AA contents were significantly higher in both experimental groups than in the CK group. These findings indicate that supplementation with *L. plantarum* accelerated fermentation stabilization and effectively preserved nutrients in strawberry vines silage. NH_3_-N in silage typically arises from protein metabolism by undesirable microorganisms (28). Elevated NH_3_-N levels not only reduce CP content but also decrease animal feed intake. In this study, NH_3_-N content was significantly higher in the CK group than in the two treatment groups. The result demonstrate that LAB inoculation effectively regulated NH_3_-N production, thereby improving the ensiling quality of strawberry vines.

Plants produce anti-nutritional factors to combat adverse environmental conditions or to regulate their nutritional composition. However, excessive levels of these anti-nutritional factors in feed can inhibit livestock growth and impair reproductive performance (29). Therefore, quantification of anti-nutritional factors becomes crucial. Tannins, as important secondary metabolites in plants, rank second only to lignin in abundance. Tuominen et al. demonstrated that the unique chemical structure of tannins renders them susceptible to decomposition under variations in temperature and pH (30). In this study, the tannin degradation rate showed a positive correlation with the relative abundance of *L. plantarum*. This correlation might be attributed either to tannin degradation by *L. plantarum* during its growth phase or to spontaneous tannin decomposition facilitated by sustained heat generation during ensiling. Additionally, Brutti et al. revealed that tannins can form complexes with CP, reducing its degradability and thereby increasing rumen-bypass CP content (31). Notably, Group I not only demonstrated significantly reduced tannin content but also maintained higher CP levels than both CK and Group II. This suggests that *L. plantarum* Z exhibits a strong capacity for protein preservation.

Long et al. reported that during the initial ensiling phase, the complex microbial communities present on silage material surfaces impede LAB from establishing dominance owing to competitive interactions with undesirable microorganisms, which may lead to an increased relative abundance of harmful species (32). As major phyla involved in silage fermentation, *Bacillota* and *Pseudomonadota* have been shown by Geddes and Rangan et al. to degrade macromolecular substances through the secretion of cellulases and hemicellulases (33, 34), with some species capable of synthesizing organic acids such as LA and AA from organic matter. The observed changes in fiber content in the present study likely correspond to the metabolic activities of these phyla. However, an excessive presence of *Pseudomonadota*, which are predominantly gram-negative bacteria, may contribute to silage spoilage and reduce aerobic stability. Throughout the ensiling process, the abundance of *Pseudomonadota* gradually declined across all groups. Bao et al. demonstrated that selected LAB strains exhibit superior environmental adaptability, enabling rapid pH reduction (35). In Group I, inoculation with *L. plantarum* facilitated the swift establishment of dominance and sustained high abundance during early ensiling. *Bacillota* abundance in Group I reached 87.26% by day 3, demonstrating the superior adaptability of *L. plantarum* to ensiling conditions.

Shafiee and Feng et al. demonstrated that strawberries and their vines contain endogenous salicylic acid (36, 37). As a precursor to salicylic acid, salicin serves as a carbon source for *L. plantarum* but not for *L. lactis*. Concurrently, Mukherjee et al. reported that *L. lactis* exhibits low tolerance to tannins, with elevated concentrations of tannic acid inhibiting its growth (38).

Group I was dominated by *Lactiplantibacillus* and *Clostridioides* during the early stages of ensiling, while Group II did not show significant *Lactobacillus* abundance. This divergence likely stems from differences in substrate utilization: unlike *Lactiplantibacillus*, *L. lactis* lacks the capacity to effectively metabolize salicin as a carbon source. Furthermore, the elevated tannin content in strawberry vines may have suppressed *L. lactis* growth, accounting for the limited abundance of this bacterium in Group II. Fang et al. reported that in high-moisture silage, low pH alone cannot completely inhibit *Clostridioides* proliferation (39), potentially leading to butyric acid fermentation and consequent losses of DM and energy. During the later stages of ensiling, both experimental groups maintained stable LA content and pH levels without significant fluctuations, suggesting that inoculation with *L. plantarum* and *L. lactis* enhanced silage stability and effectively controlled *Clostridioides* proliferation. Although initially lower in abundance, *L. lactis* populations remained stable over the prolonged ensiling period. Additionally, *Klebsiella* abundance gradually decreased over time, with *Lactiplantibacillus* relative abundance remaining predominant in Group II by day 45.

DMI, ADG, and F/G are key indicators for evaluating growth performance in meat sheep (40). Volatile fatty acids (VFAs) in the rumen serve as primary energy sources for ruminants and play crucial roles in rumen health and energy metabolism. In this study, supplementation with LAB-fermented silage resulted in a significantly higher ADG and lower F/G relative to the CK group, although no significant differences were observed in DMI. This phenomenon may be attributed to the increased levels of LA and AA in the silage-supplemented diets, which directly contributed to enhanced energy supply for Hu sheep. The reduction in tannin content through ensiling likely lowered it below the threshold that inhibits DMI (41), while physiological adaptations in small ruminants, including increased salivary secretion, helped mitigate tannin-induced effects. These findings align with those of Lin et al. (42), who reported that a tannin concentration of 0.2% maintained stable DMI while enhancing ADG and reducing F/G by suppressing excessive ruminal protein degradation and improving intestinal amino acid absorption efficiency. In the present study, tannin content in the strawberry vines of Group I was significantly reduced compared to Group II (*p* < 0.05), a change attributed to *L. plantarum* supplementation. This reduction in tannin attenuated its inhibitory effects on the growth performance in Hu sheep. Although Group I exhibited numerically higher ADG and lower F/G than Group II, these differences were not statistically significant (*p* > 0.05).

Emkani et al. demonstrated that LAB fermentation alters the spatial configuration of dietary proteins, thereby significantly enhancing CP apparent digestibility (43). Rumen microorganisms convert fibrous components into VFAs through fermentation, serving as vital energy sources. Hristov et al. reported that ensiling markedly improves fiber apparent digestibility (44), which is attributed to acid-induced softening of fiber structures and limited cellulase secretion by microbial activity. Besharati et al. suggested that tannin-nutrient complexes might influence digestibility, with low tannin concentrations potentially enhancing CP apparent digestibility in ruminants (45). The present experiment revealed synergistic effects between low tannin levels and silage microorganisms, including (1) promotion of DM and NDF metabolism; and (2) improved CP digestibility via the combined action of microbial activity and optimized tannin concentration. Furthermore, the reduced tannin content in Group I, mediated by *L. plantarum* activity, was associated with numerically higher apparent digestibility of NDF and CP compared to Group II, although these differences were not statistically significant (*p* > 0.05).

Wen et al. demonstrated that silage supplementation significantly improves apparent digestibility in ruminants without causing notable alterations in microbial composition at either the phylum or genus levels (46). In the present study, *Bacteroidota* and *Bacillota* showed significant differences among the three groups. Dao et al. identified *Prevotella* as a keystone genus involved in carbohydrate and hydrogen metabolism in ruminants (47). These cellulose-degrading genera (*Prevotella*, *Bacteroides*, and *Clostridium*) secrete cellulases and hemicellulases, which hydrolyze structural polysaccharides into soluble sugars and oligosaccharides, thereby enhancing feed digestibility. Additionally, *Bacteroides* contributes to amino acid synthesis, supplying substrates for microbial protein production. *Capnocytophaga* exhibits proteolytic activity, degrading dietary proteins and thereby improving CP digestibility through microbial protein synthesis. Additionally, this genus modulates host immune responses by attenuating inflammatory reactions and promoting animal health. All three genera—*Bacteroides*, *Capnocytophaga*, and *Clostridium*—are involved in the synthesis of short-chain fatty acids, which serve as energy sources for ruminants. Group II showed moderate increases in the abundances of *Bacteroides*, *Capnocytophaga*, and *Clostridium* relative to the CK group. Notably, the CK group displayed a significantly higher abundance of *Clostridium* than Group I.

In this study, inclusion of strawberry vine silage in Group I markedly reduced the relative abundances of the predominant ruminal genera Clostridium and Ercella. Both Ercella and Clostridium belong to the order Clostridiales, which play multiple roles in anaerobic digestion, including high rates of cellulose hydrolysis, protein catabolism, and acidogenesis, leading to the production of short-chain fatty acids, CO₂, and H₂ (48). As a dynamic and balanced system, the rumen microbiota may exhibit a reduced abundance of Ercella in Group I either because the improved pre-digestion effect lowered the rumen’s functional demand for this genus, or because the increased abundance of other genera compensated for this functional niche. Lengowski et al. reported that variations in the nutritional composition of different silages can influence the structure of the ruminal microbial community (49). Furthermore, other studies have demonstrated that microorganisms present in silage may contribute to alterations in the ruminal microbiota (50). However, the reductions in these two genera observed in Group I Hu sheep were not detected in the strawberry vine silage itself, suggesting that shifts in ruminal microbial composition were primarily attributable to differences in the nutritional structure of the feed ingredients rather than the direct introduction of exogenous microbes. Collectively, these findings indicate that supplementation with strawberry vine silage supports rumen microbial stability in Hu sheep.

## 5 Conclusion

Two LAB strains, *L. plantarum* and *L. lactis*, were isolated from strawberry vines. *L. plantarum* exhibited greater carbon source utilization and tannin-degrading capacity than *L. lactis*, resulting in a more pronounced pH reduction during the early stage of ensiling in the *L. plantarum* treatment group, which indirectly improved the silage quality of strawberry vines. When both strains were used for ensiling and the resulting silages were fed to Hu sheep, neither *L. plantarum* nor *L. lactis* was detected in the rumen microbiota via 16S rDNA analysis. However, compared with the CK group, *L. plantarum* treatment reduced the relative abundance of the dominant rumen genera *Clostridium* and *Ercella*. Apparent digestibility of DM, NDF, and CP was increased in the *L. plantarum* group, whereas improvements in DM and CP digestibility were observed in the *L. lactis* group. Both treatments increased ADG and decreased F/G in Hu sheep.

## Data Availability

The original contributions presented in the study are publicly available. This data can be found here: https://www.ncbi.nlm.nih.gov/ PRJNA1335296, PRJNA1335361.
